# Supplementary material for: Effects of Different Feeding Methods on the Structure, Metabolism, and Gas Production of Infant and Toddler Intestinal Flora and Their Mechanisms
Source: Nutrients. 2022 Apr 9;14(8):1568. doi: 10.3390/nu14081568 (PMC9027170; doi:10.3390/nu14081568)
Supplement: Supplementary file 1 [file nutrients-14-01568-s001.zip › nutrients-1656933-supplementary.pdf]

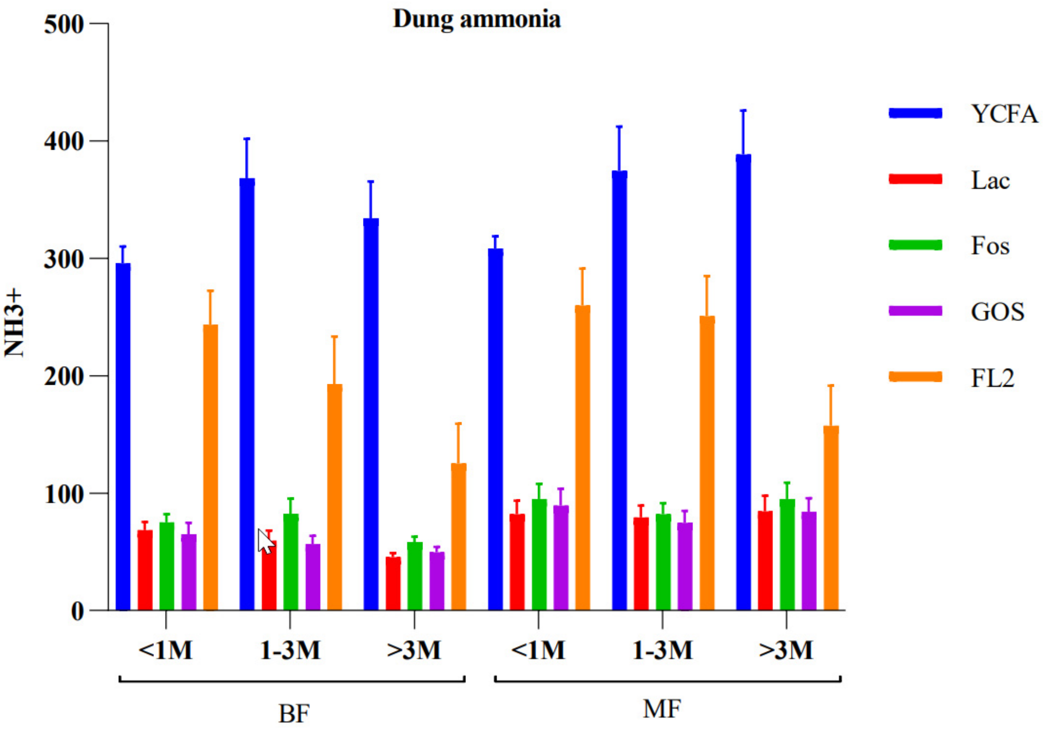

Figure S1. Comparison of *in vitro* simulated fermented fecal ammonia levels in infants and toddlers fed differently and at different growth stages

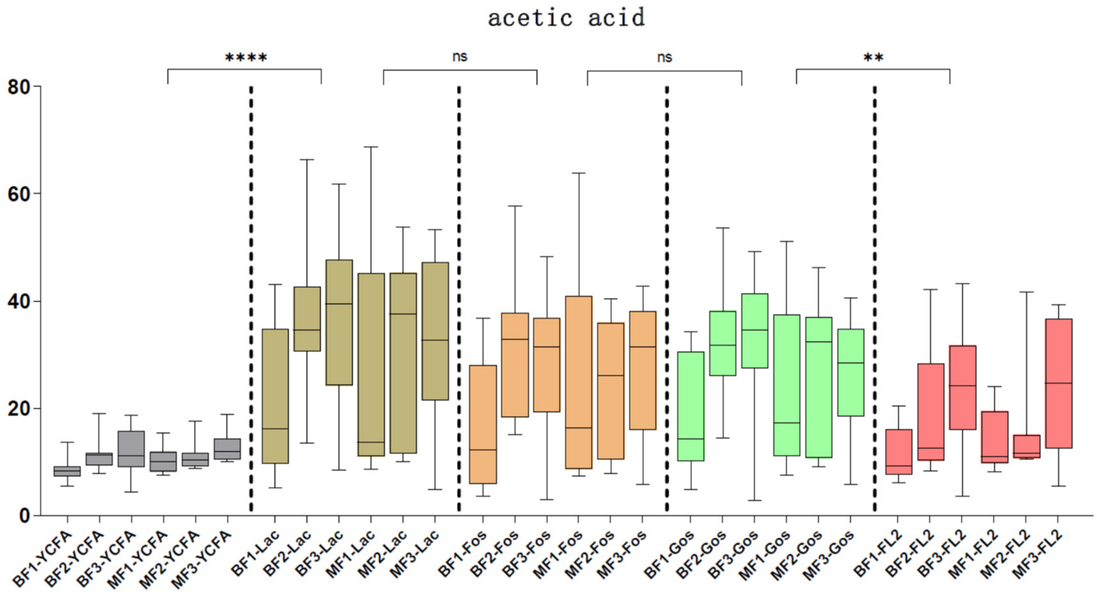

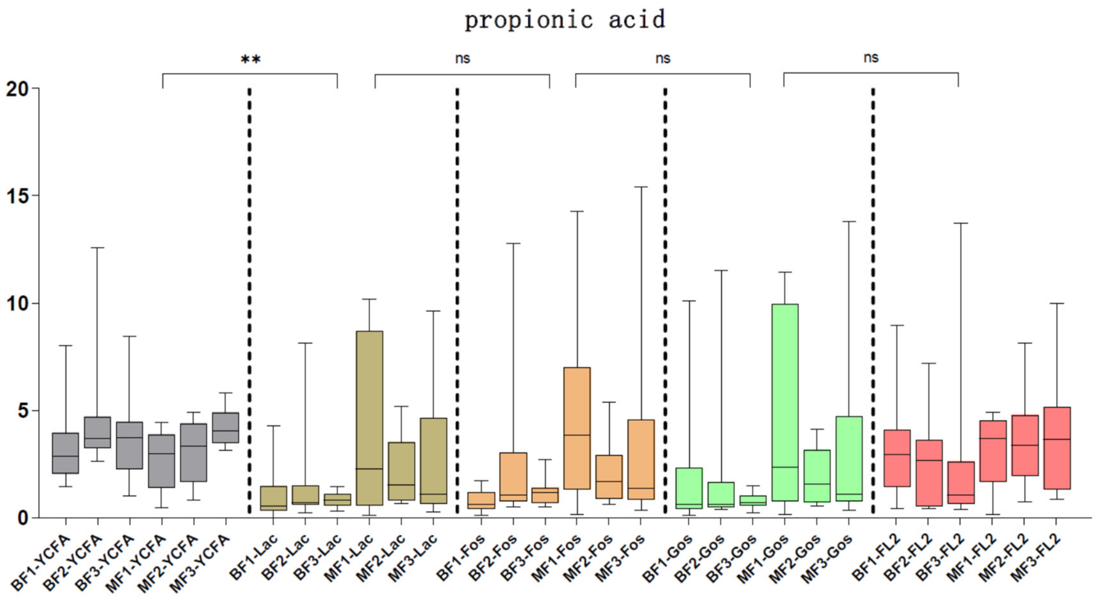
propionic acid
**
ns
ns
ns
20
15
10
5
0
BF1-YCFA
BF2-YCFA
BF3-YCFA
MF1-YCFA
MF2-YCFA
MF3-YCFA
BF1-Lac
BF2-Lac
BF3-Lac
MF1-Lac
MF2-Lac
MF3-Lac
BF1-Fos
BF2-Fos
BF3-Fos
MF1-Fos
MF2-Fos
MF3-Fos
BF1-Gos
BF2-Gos
BF3-Gos
MF1-Gos
MF2-Gos
MF3-Gos
BF1-FL2
BF2-FL2
BF3-FL2
MF1-FL2
MF2-FL2
MF3-FL2

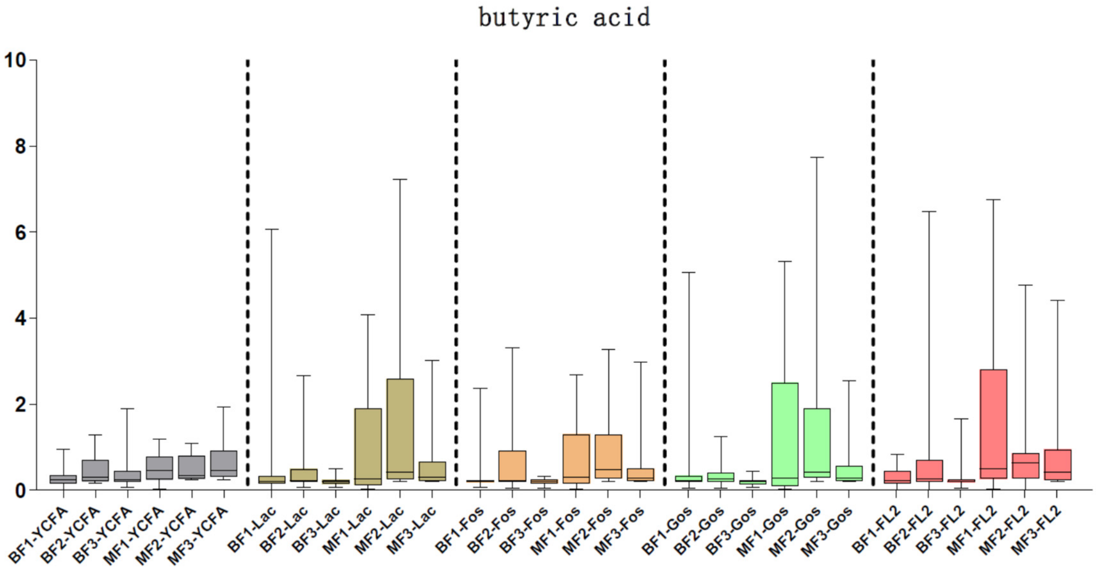
butyric acid
10
8
6
4
2
0
BF1-YCFA
BF2-YCFA
BF3-YCFA
MF1-YCFA
MF2-YCFA
MF3-YCFA
BF1-Lac
BF2-Lac
BF3-Lac
MF1-Lac
MF2-Lac
MF3-Lac
BF1-Fos
BF2-Fos
BF3-Fos
MF1-Fos
MF2-Fos
MF3-Fos
BF1-Gos
BF2-Gos
BF3-Gos
MF1-Gos
MF2-Gos
MF3-Gos
BF1-FL2
BF2-FL2
BF3-FL2
MF1-FL2
MF2-FL2
MF3-FL2

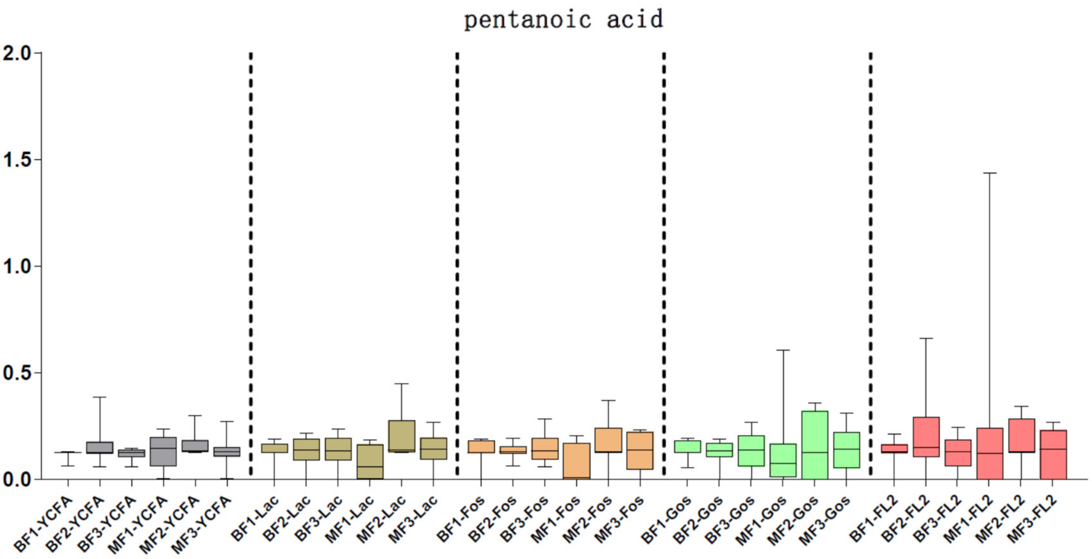
pentanoic acid
2.0
1.5
1.0
0.5
0.0
BF1-YCFA
BF2-YCFA
BF3-YCFA
MF1-YCFA
MF2-YCFA
MF3-YCFA
BF1-Lac
BF2-Lac
BF3-Lac
MF1-Lac
MF2-Lac
MF3-Lac
BF1-Fos
BF2-Fos
BF3-Fos
MF1-Fos
MF2-Fos
MF3-Fos
BF1-Gos
BF2-Gos
BF3-Gos
MF1-Gos
MF2-Gos
MF3-Gos
BF1-FL2
BF2-FL2
BF3-FL2
MF1-FL2
MF2-FL2
MF3-FL2

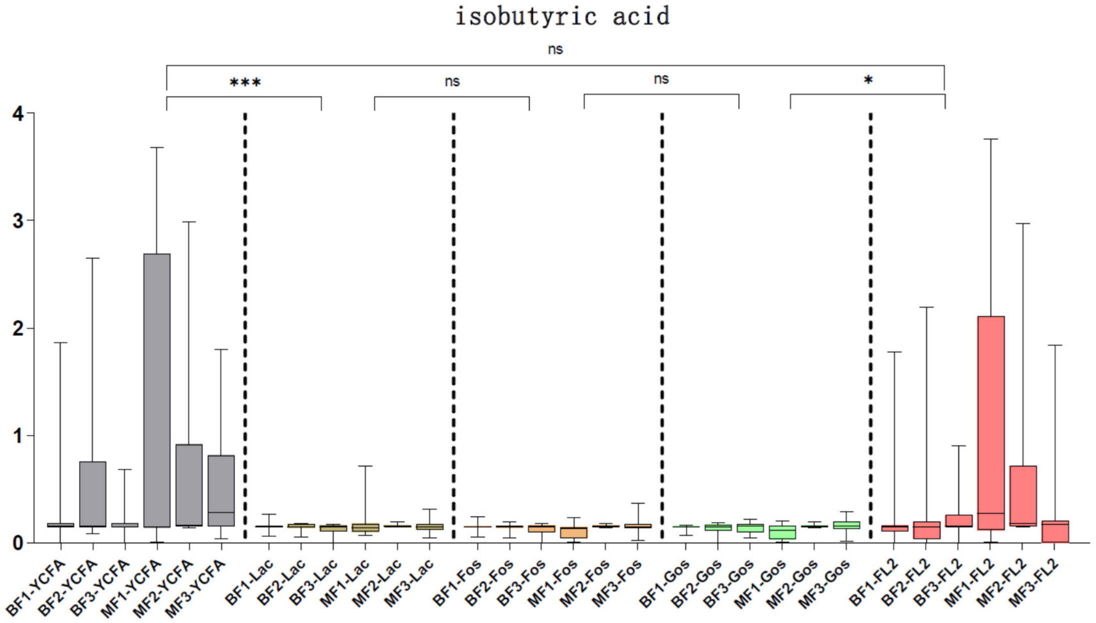
isobutyric acid
ns
***
ns
ns
*
4
3
2
1
0
BF1-YCFA
BF2-YCFA
BF3-YCFA
MF1-YCFA
MF2-YCFA
MF3-YCFA
BF1-Lac
BF2-Lac
BF3-Lac
MF1-Lac
MF2-Lac
MF3-Lac
BF1-Fos
BF2-Fos
BF3-Fos
MF1-Fos
MF2-Fos
MF3-Fos
BF1-Gos
BF2-Gos
BF3-Gos
MF1-Gos
MF2-Gos
MF3-Gos
BF1-FL2
BF2-FL2
BF3-FL2
MF1-FL2
MF2-FL2
MF3-FL2

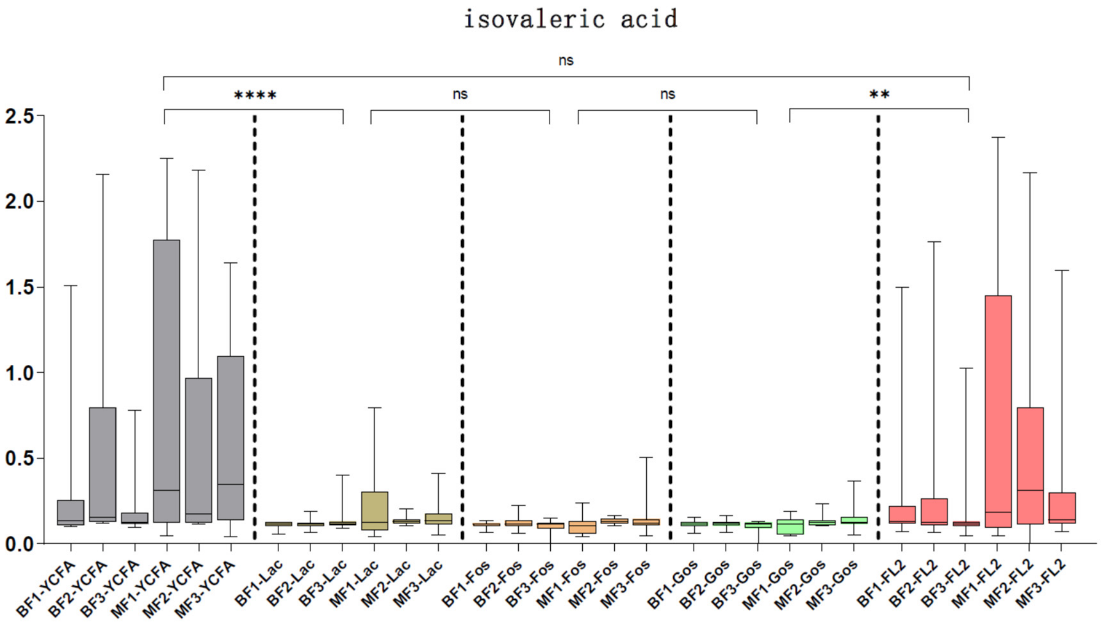

Figure S2. Comparison of *in vitro* simulated fermentation of SCFA production by fecal bacteria from infants and toddlers fed differently and at different growth stages

Data were expressed as the mean ± SEM. Differences are considered significant at $P < 0.05$ (*), $P<0.01$ (**), $P<0.001$ (***) and $P < 0.0001$ (****)

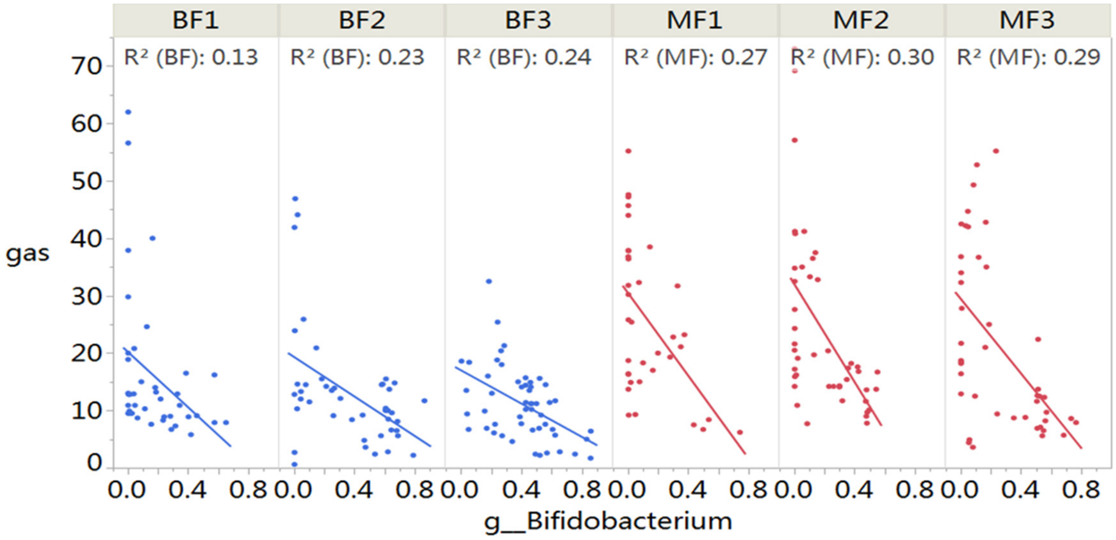

Figure S3 Correlation analysis between *in vitro* fermentation gas production and abundance of *Bifidobacterium* spp. in infants and toddlers fed differently and at different growth stages

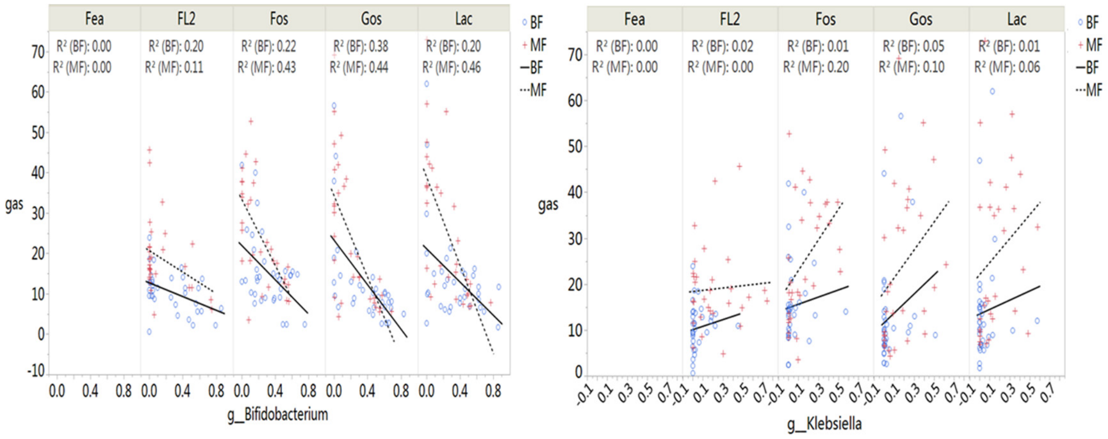

Figure S4 Correlation analysis between fermentation gas production and abundance of *Bifidobacterium* and *Klebsiella* in fecal bacteria and carbon sources in media from different feeding methods

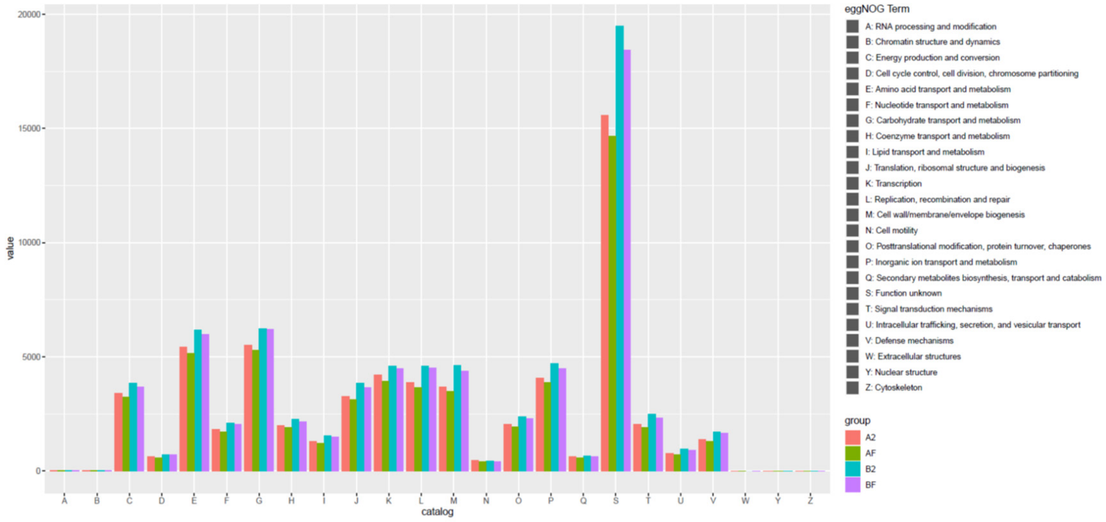

Figure S5. Analysis of differences in functional gene enrichment in fecal bacteria from different feeding methods grown on media with different carbon sources (2'-FL and FOS). A2 represent the fermentation of intestinal flora from MF in the medium containing the carbon source of 2'-FL; AF represent the fermentation of intestinal flora from MF in the medium containing the carbon source of FOS; B2 represent the fermentation of intestinal flora from BF in the medium containing the carbon source of 2'-FL; BF represent the fermentation of intestinal flora from BF in the medium containing the carbon source of FOS.

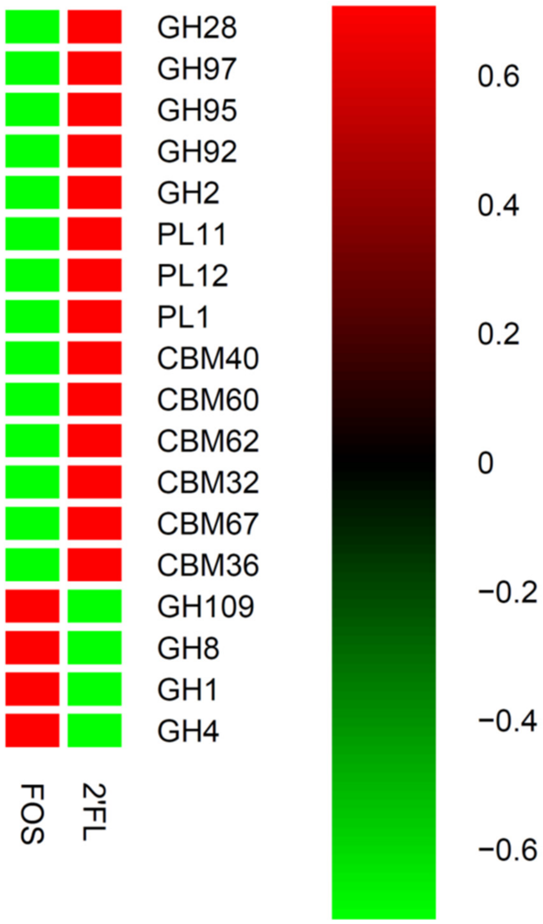

Figure S6. The heat map of the CAZY families showing the enrichment of genes related to oligosaccharide metabolism in infant fecal bacteria cultured in medium with two different carbon sources (2'-FL and FOS).

Table S1. The results of transcriptome analysis

Group	log2FoldChange	pvalue	padj	gene_name	gene_biotype	gene_description
Kpn vs 2FL Kpn	-4.21	2.10E-02	1.00E+00	SERF1A	protein_coding	small EDRK-rich factor 1A [Source:HGNC Symbol;Acc:HGNC:10755]
Kpn vs 2FL Kpn	-4.26	2.56E-02	1.00E+00	MARK2P17	processed_pseudogene	microtubule affinity regulating kinase 2 pseudogene 17 [Source:HGNC Symbol;Acc:HGNC:50318]
CK vs Kpn	5.707077	0.0098251	1	MARK2P17	processed_pseudogene	microtubule affinity regulating kinase 2 pseudogene 17 [Source:HGNC Symbol;Acc:HGNC:50318]
CK vs Kpn	4.193578	0.0160419	0.933202	SERF1A	protein_coding	small EDRK-rich factor 1A [Source:HGNC Symbol;Acc:HGNC:10755]
Cp vs 2FL Cp	0.80	3.24E-04	1.00E+00	TRIB3	protein_coding	tribbles pseudokinase 3 [Source:HGNC Symbol;Acc:HGNC:16228]
Cp vs 2FL Cp	1.01	1.16E-02	1.00E+00	ADM2	protein_coding	adrenomedullin 2 [Source:HGNC Symbol;Acc:HGNC:28898]
Cp vs 2FL Cp	0.48	1.43E-02	1.00E+00	PCK2	protein_coding	phosphoenolpyruvate carboxykinase 2, mitochondrial [Source:HGNC Symbol;Acc:HGNC:8725]
CK vs Cp	-2.7753	0.0061879	0.999979	ADM2	protein_coding	adrenomedullin 2 [Source:HGNC Symbol;Acc:HGNC:28898]

CK vs Cp	-2.37877	0.0157859	0.999979	TRIB3	protein_coding	tribbles pseudokinase 3 [Source:HGNC Symbol;Acc:HGNC:16228]
CK vs Cp	-1.50417	0.0346799	0.999979	PCK2	protein_coding	phosphoenolpyruvate carboxykinase 2, mitochondrial [Source:HGNC Symbol;Acc:HGNC:8725]
